# Examining associations among daily discrimination, psychosocial risk factors, and pain outcomes in people with chronic low back pain

**DOI:** 10.3389/fpain.2025.1531187

**Published:** 2026-01-12

**Authors:** Joanna M. Hobson, Matthew C. Morris, Robert E. Sorge, D. Leann Long, Tammie Quinn, Demario S. Overstreet, Asia M. Wiggins, Eeshaan K. Bajaj, Jonas G. Dembowski, Edwin N. Aroke, Burel R. Goodin, Calia A. Torres

**Affiliations:** 1 University of Florida, Community Dentistry and Behavioral Sciences, Gainesville, FL, United States; 2 Department of Psychiatry and Human Behavior, University of Mississippi Medical Center, Jackson, MS, United States; 3 Department of Anesthesiology, Vanderbilt University Medical Center, Nashville, TN, United States; 4 Department of Biostatistics, School of Public Health, University of Alabama at Birmingham, Birmingham, AL, United States; 5 School of Medicine, University of Alabama at Birmingham, Birmingham, AL, United States; 6 School of Nursing, University of Alabama at Birmingham, Birmingham, AL, United States; 7 Department of Anesthesiology, Washington University, St. Louis, MI, United States; 8 Gulfshore Behavioral Health, Fort Myers, FL, United States

**Keywords:** discrimination, sleep, depressive symptoms, low-back pain, stress

## Abstract

**Introduction:**

Substantial evidence suggests that experiences of discrimination negatively influence sleep, depressive symptoms, stress, and pain. The purpose of this study was to evaluate the strength of the associations between discrimination and pain, and to determine which psychosocial risk factors help explain these associations.

**Methods:**

Participants (*N* = 208) underwent two study sessions, where they completed the Everyday Discrimination Scale, Perceived Stress Scale, Centers for Epidemiological Studies Depression Scale, Insomnia Severity Index, and the Brief Pain Inventory-Short Form. Demographic data was also obtained from participants.

**Results:**

Majority of the participants self-identified as women (55.3%), and Black (62%). There were positive associations between discrimination and insomnia (*p* < .001), depressive symptoms (*p* < .001), perceived stress (*p* < .001), pain severity (*p* < .001) and pain interference (*p* < .001). Hierarchical regressions showed that identifying as Black (*p* < .001), having greater depressive symptoms (*p* = .03), and greater insomnia symptoms (*p* < .001) were associated with greater pain severity in the past 24 h. Similarly, older age (*p* = .01), identifying as Black (*p* = .002), having lower education (*p* = .04), taking medications (*p* = .04), greater depressive (*p* < .001) and insomnia symptoms (*p* < .001) were associated with greater pain interference. The indirect effect of discrimination on pain severity was significant (*β* = .015, Bootstrap 95% CI.003–.030). Additionally, there was a significant indirect effect of discrimination on pain interference (*β* = .015, Bootstrap 95% CI.004–.031). Exploratory models showed an indirect effect of pain severity (*β* = .014, Bootstrap 95% CI.001–.029) and interference (*β* = .012, Bootstrap 95% CI.000 to .029) on discrimination via psychosocial risk factors.

**Discussion:**

Our findings highlight the harmful associations between discrimination, mental health outcomes, pain severity, and reduced quality of life. Additionally, these findings emphasize the need for more stress engaged research to continue exploring these potential relationships, identify cause-effect and inform the development of future interventions focused on reducing the negative impact of stress on pain outcomes – especially for minority groups who are disproportionately affected by pain disparities.

## Introduction

Chronic low back pain (cLBP) is one of the most common and debilitating conditions in the U.S. with a prevalence of 40.9%, amounting to 20.5 million people (1–3). cLBP is defined as pain lasting at least 3 months or longer and is one of the top five reasons for primary care visits (4). Most cLBP conditions are non-specific, meaning there is no clear pathology of the spine or related tissue injuries (4). Without a clear target, treatment for cLBP can be difficult and stressful for the individuals experiencing pain. In fact, cLBP patients continuously seek optimal treatment without significant results, and these experiences can trigger stigma, which perpetuates discrimination due to their condition (5). Specifically, people living with cLBP have reported experiences of stigma and discrimination in healthcare settings and their communities, such that physicians may assume that they “just want opioids”, and they're considered “lazy” in their communities (6–9). If they're also a person of color, there is an additional assumption of substance abuse in healthcare settings that warrants attention, leading to the misdiagnosis and treatment of pain (10, 11).

Experiences of discrimination occur in a continuum, and across different contexts (12–14). Discrimination can be presented covertly or overtly and is perceived as a social threat given the very real possibility for physical assault, verbal abuse, ostracism, and exclusion among minoritized groups (15, 16). According to Erving Goffman, stigma is a belief or thought about a particular individual or group that is centered around power and control and is used to perpetuate various forms of discrimination (e.g., racial, weight-related, health-condition, gender identity) (17). These forms of discrimination ultimately promote inequalities and inequities, such as reduced social acceptance, reduced opportunities (due to unequal resources), and in the worst cases, police brutality (18, 19). It is widely documented that inequities in pain management are constantly reiterated as healthcare providers under-diagnose and under-treat chronic pain, especially in marginalized populations (e.g., people of color, women, older individuals, people living with HIV) (10, 20–22). In addition to experiences of discrimination from healthcare providers, people with cLBP may experience different forms of stigma and discrimination from their communities (6, 7). For example, one qualitative study found that individuals who experience cLBP have been deemed as “lazy”, “poor”, or “just wanting to get out of work”. The same article went on to discuss how the pain isn't specific or due to injury, which makes it hard for people to understand (6). The amount of pressure that's needed to adapt from discriminatory experiences can often influence high-effort coping (e.g., feeling that you have to work harder due to racial or other-related discrimination)—and this experience, whether positive or negative, can contribute to a variety of health conditions (high blood pressure, pain development), as cited in previous work (23–25). These findings have been highlighted in the minority stress theory and the weathering hypothesis (26–29). The minority stress theory posits that minorities are disproportionately affected by stigma and discrimination, and in turn are exposed to adverse mental and physical health conditions (26, 30). The weathering hypothesis expands upon the minority stress theory, by showing that Black or African American people age at quicker rates than Caucasians due to disproportionate stress and coping mechanisms (27, 28, 31). Thus, understanding the mechanisms by which discrimination impacts pain is warranted. While psychological distress has been studied as a potential link between discrimination and pain (32, 33); it is likely that a combination of psychological and physiological vulnerabilities are at play, and all contribute to the negative effects of discrimination on pain outcomes.

Two major stress response systems are activated once discrimination is perceived, including the hypothalamic-pituitary-adrenal (HPA) axis, which releases stress-related hormones such as cortisol, and the autonomic nervous system (ANS), which releases pro-inflammatory cytokines, T-cells, and B-cells (34, 35). When these processes become chronic, they can contribute to poor physical and mental health conditions (36). Chronic activation of the salience network—or a system that responds to and receives homeostatic input—can contribute to HPA-axis dysregulation, hypervigilance, anxiety and depressive disorders, and chronic pain (35, 37, 38). Pain and stress have also been conceptualized as “two distinguished yet overlapping processes presenting multiple conceptual and physiological overlaps” (33). Taken together, the physiological impact of discrimination is multifaceted, can be deemed as stressful, and could leave vulnerable populations at risk for co-morbid physical and mental conditions.

Discrimination based on race, gender, sexual orientation, religion, weight, and education levels has known effects on the health and well-being of minoritized individuals (8, 39–43). Prior studies have found that experiences of discrimination have direct effects on sleep, depressive symptoms, and pain (44–46). Specifically, individuals who experience a greater frequency of stigma report poorer sleep, more depressive symptoms, and greater pain than those with lower experiences of stigma. There is a bidirectional association between pain and sleep, such that sleep is often a potent influencer on pain than pain is on sleep (47, 48). Accounting for the effect of discrimination on these relationships may help elucidate how negative and stressful experiences due to discrimination could lead to insomnia symptoms and worse pain outcomes.

The mechanisms by which discrimination affects pain-related outcomes and sleep may be due, in part, to the effect of stress on endogenous pain regulation (49, 50). Endogenous pain regulation involves the brain's “pain matrix” or regulatory systems that control the perception of nociceptive events (51, 52). Given the high rates of depression experienced by people with cLBP (53–55), and the negative effects of discrimination on pain processing, we will begin to identify the potential influence of discrimination on negative pain-related outcomes. Therefore, our primary aim is to determine whether the associations between discrimination and pain outcomes (i.e., pain severity and interference) can be partially explained by psychosocial risk factors (i.e., insomnia, perceived stress, and depressive symptoms). Our exploratory aim is to elucidate the indirect effects of pain severity and interference on discriminatory experiences. We hypothesize that a higher frequency of discriminatory experiences will be associated with worse pain outcomes through greater perceived stress, greater depressive symptoms, and poorer sleep. Specifically, perceived stress, depressive symptoms, and poor sleep would sequentially mediate the association between experiences of discrimination, pain severity and pain interference. Additionally, there will be a significant indirect effect of pain severity and interference on daily discrimination. To our knowledge, this is the first study to measure the simultaneous effects of discrimination and psychosocial risk factors (i.e., stress, depression, insomnia) on pain among individuals with cLBP, and to test the proposed reverse mediation pathways. These findings will set the foundation for future stress-based intervention research, bridging the gap between social and environmental stressors, and pain disparities in minoritized groups.

## Methods

### Study overview

This study was a part of a parent project investigating ethnic/racial and socioeconomic differences in chronic low back pain (ERASED) (56, 57). The parent project examined racial differences in biopsychosocial factors that influence pain. The data in this study were collected between November 2017 and July 2022. Interested participants completed a telephone screening to determine eligibility, and two laboratory-based sessions spaced one week apart. Questionnaires assessing depressive and insomnia symptoms, and perceived stress were completed in week 1, and questionnaires assessing pain severity and interference were completed at weeks 1 and 2. For this study, we used the BPI-SF scores that were completed at week 2. Total compensation for the completion of both experimental sessions was $400. Specifically, participants received $150 per session, $60 for the completion of at-home sleep monitoring, and $10 for each follow-up telephone call. Inclusion criteria were as follows: (1) between the ages of 18–85; (2) able to read, write, and comprehend English, (3) self-identify as Black/African American or White/Caucasian, and (4) report low back pain for 3 consecutive months that was present for at least half of the days in the past 6 months, with the primary complaint being non-specific low back pain. Exclusion criteria were as follows: (1) low back pain attributed to other factors such as ankylosing spondylitis, infection, malignancy, or compression fracture, (2) surgical intervention or accident/trauma in the past year, (3) presence of any systemic rheumatic conditions, (4) uncontrolled hypertension, cardiovascular, or peripheral artery disease, (5) poorly controlled diabetes, (6) neurological disease, (7) psychiatric hospitalization within the past year, and (6) pregnancy. Written informed consent was given to the participant and signed. This study was approved by the local IRB.

### Participants

Participants in the Birmingham, Alabama community were recruited via flyers posted at the UAB Pain Treatment clinic and surrounding areas. A total of 208 participants were recruited for this study. Full demographic details will be presented in Table 1.

**Table 1:** Participant characteristics (*N* = 208).

Variable (Mean, SD or *N*, %)	
Demographics	(Mean, SD or *N*, %)
Age	44.94 (14.37)
Gender, *N* (%)	
Men	93 (44.7%)
Women	115 (55.3%)
Race, *N* (%)	
African American/Black	129 (62.0%)
Caucasian/White	79 (38.0%)
Income*	
Lower income (below 30,000)	83 (41.7%)
Higher income (above 30,000)	116 (58.3%)
Education	
Some college experience	162 (77.9%)
No college experience	46 (22.1%)
Medications & comorbidities	
Medications*	
Taking medications	165 (81.3%)
Not taking medications	38 (18.7%)
Medication Type*	
Opioids	18 (8.7%)
NSAIDs	89 (42.8%)
Muscle relaxers	19 (9.1%)
Antidepressants	33 (15.9%)
Neuroleptics	3 (1.4%)
Benzodiazepines	6 (2.9%)
Vitamins	35 (16.8%)
Botanicals	5 (2.4%)
Blood pressure medication	48 (23.1%)
Diabetic medication	14 (6.7%)
Allergy medication	10 (4.8%)
Thyroid medication	9 (4.3%)
Cholesterol medication	10 (4.8%)
Non-NSAID Pain medication	28 (13.5%)
Anticonvulsant medication	24 (11.5%)
Asthma medication	7 (3.4%)
Comorbidities*	
High blood pressure	70 (33.7%)
Heart disease	3 (1.4%)
Cancer	0
Diabetes (Hba1c > 7%)	23 (11.1%)
Ankylosing Spondylitis	1 (0.5%)
Infection	0
Parkinson's Disease	0
Multiple Sclerosis	0
Epilepsy	1 (0.5%)
Syndromic obesity	0
Stroke	0
Seizure	1 (0.5%)
Rheumatoid arthritis	3 (1.4%)
Lupus erythematosus	0
Fibromyalgia	0
Major depression/BPD	19 (9.1%)
Other mental health condition	36 (17.3%)
HIV	0
Survey Characteristics	
TEDS—Discrimination, Mean (SD)*	9.78 (8.70)
PSS—Stress, Mean (SD)	21.61 (3.88)
CES-D—Depressive Symptoms, Mean (SD)	16.63 (10.64)
ISI—Insomnia Symptoms, Mean (SD)	12.32 (6.98)
BPI-SF Pain Severity Mean (SD)	4.47 (2.35)
BPI-SF Pain Interference Mean (SD)	3.10 (2.46)

BPI-SF, brief pain inventory short form; CES-D, center for epidemiological studies depression scale; ISI, insomnia severity index; PSS, perceived stress scale; TEDS, the everyday discrimination scale.

* Has missing data.

### Measures

#### Demographic Questionnaire

The cLBP demographic questionnaire is a 22-item questionnaire that assesses participants' race, gender, income levels, and education amongst other characteristics. Participants were allowed to circle one or more responses regarding race, choosing from the following: Black, White, Asian, American Indian, Native Hawaiian and Multiracial. Similar responses were recorded for other characteristics. Data were coded such that variables of interest were 1 (e.g., Black/African American race, Male gender, no college experience, lower income) and other reference groups were coded as 0. Income level was determined based on the average income in Alabama for 2023, and those who fell below the average income were considered lower income. This scale was developed in our lab, and has been used in previous studies (56, 57).

#### Medications

On a separate cLBP screening questionnaire assessing pain and comorbid conditions, participants were asked the following question: “Are you currently taking any medications (prescription or over the counter) for pain or any other reason?”. Responses to this question were coded as yes (1) or no (0), and followed up with a list of the types of medications, ranging from the following options: opioids, non-steroid anti-inflammatory drugs (NSAIDS), muscle relaxers, antidepressants, neuroleptics, benzodiazepines, vitamins, botanicals, and blood pressure medication. For this study, we used the dummy-coded variable that assessed whether people took prescriptions or over-the-counter medications. However, the descriptive statistics for pharmacological treatments used on this scale will be shown below. Lastly, because this scale was developed in our lab previously, it has not been used in prior studies. Thus, data interpretation should be conducted with caution.

#### The Everyday Discrimination Scale (TEDS)

The Everyday Discrimination Scale (TEDS) is a 9-item measure that assesses the frequency of discriminatory experiences and unfair treatment in an individual's day-to-day life. Scores on this scale are summed on a 5-point Likert scale (0 = Never to 5 = Almost every day), and range from 0 to 45, with higher scores suggesting a greater frequency of discrimination. TEDS measures the frequency of the following events: being treated with less courtesy than others, less respect than others, receiving poorer service than others in restaurants or stores, people acting as if you're not smart, acting as if they're better than you, afraid of you, or think you're dishonest, being called names or insulted, and being threatened or harassed. Participants are then asked a follow-up question if they answered “A few times a year” or “more frequently” to at least one question, which addresses the reasoning for those experiences (e.g., based on race, gender, age, weight, and etc.) Afterwards, participants are asked to enter an open response in the section labeled “Other” to explain reasons that may not be listed (58–60). This scale demonstrated high internal consistency for our study (Cronbach's *α* = .90) and has shown high reliability and validity in prior studies, with Cronbach's *α* ranging from 0.74–0.87 (58, 61).

#### Perceived Stress Scale (PSS)

The Perceived Stress Scale (PSS) is a 10-item scale that assesses daily experiences and perceptions of stress. This scale assesses daily hassles, stressors, and major life events in the last month, and was originally developed to measure an individual's perception of stressful life events (e.g., discrimination). This measure has demonstrated high reliability and internal validity in multiple groups (e.g., community dwelling adults, college students, people with psychiatric conditions), with Cronbach's alpha being above 0.80 for most studies (62–65). Scores on this scale are obtained by reverse coding items 4, 5, 7 & 8 and summing across all scale items. Additionally, scores on this scale range from 0 to 40, with 0–13 indicating low stress, 14–26 indicating moderate stress, and 27–40 indicating high perceived stress. This scale also had good internal consistency and reliability in our study (Cronbach's *α* = .87).

#### Center for Epidemiological Studies Depression Scale (CES-D)

The Center for Epidemiological Studies Depression Scale (CES-D) is a 20-item measure that assesses the frequency of depressive symptoms in the past week (66, 67). Scores on the CES-D range from 0 to 60, with responses ranging from 0 (never or rarely) to 3 (most of the time), and higher scores representing a greater severity of depression. A cut-off score of 16 or more on this scale indicates a possibility for clinical depression (68). Depressive symptoms that are assessed by the CES-D include: negative mood, guilt/worthlessness, helplessness/hopelessness, psychomotor retardation, loss of appetite, and sleep disturbances. This scale has demonstrated high validity and reliability in the general population (Cronbach's *α* = 0.85), single Black mothers (Cronbach's *α* = 0.81), chronic pain patients (Cronbach's *α* ranging from 0.84 to 0.90) and people with HIV (Cronbach's *α* = 0.92) (66, 69–72). We reverse coded items 4, 8, 12, and 16 for this scale, and summed all 20 items to get total scores. Lastly, the CES-D demonstrated high internal consistency and reliability for our study (Cronbach's *α* = .90).

#### Insomnia Severity Index

The Insomnia Severity Index (ISI) is a 7-item questionnaire that assesses clinically significant symptoms of insomnia (73). Scores on the ISI were summed to get a total score, and ranged from 0 to 28, with higher scores indicating a greater severity of insomnia symptoms. Interpretation of scores for this measure were as follows: 0–7 = no clinically significant insomnia; 8–14 = subthreshold insomnia; 15–21 = moderate insomnia; 22–28 = severe insomnia (74). For this study, however, the ISI was analyzed as a continuous variable to examine the severity of symptoms. The ISI index has been deemed reliable and valid, detecting cases of insomnia in the general population and those with sleep symptoms (Cronbach's *α* ranging from 0.90 to 0.91) and people with clinically significant insomnia (Cronbach's *α* = 0.74) (73, 74). The ISI demonstrated excellent internal consistency and reliability in our study (Cronbach's *α* = .91).

#### Pain Severity & Interference

The Brief Pain Inventory Short-Form (BPI-SF) is an 11-item pain scale that measures pain severity, and how pain interferes with daily functioning (75). This scale includes a 4-item severity scale, and a 9-item interference scale that are each averaged to form two composite scores—pain severity and pain interference (76). Pain severity was assessed by averaging the 4 items assessing the current, worst, least, and average amount of pain in the past 24 h, with each item that ask how pain interfered with general activity, mood, sleep, mobility, work (inside and outside of the home), your relationships with others, and enjoyment of life in the past 24 h. Higher scores on the BPI-SF indicate greater pain severity or interference. The BPI-SF has demonstrated high reliability and validity in patients with chronic neuropathic pain and musculoskeletal pain (Cronbach's *α* = 0.90 and above) (75, 77). The BPI-SF also demonstrated excellent internal consistency and reliability for the pain severity (Cronbach's *α* = .92) and interference (Cronbach's *α* = .95) subscales in our study.

### Data analysis & handling

All analyses were performed using SPPS v.28 (IBM, Corp.). According to prior theory, listwise deletion can be used when less than 15% of data are missing, and data appears to be missing at random (78). Given these circumstances, we used listwise deletion to conduct our analyses. Normality tests were also run to determine whether parametric or non-parametric analyses should be employed. Apart from the Insomnia Severity Index and the Pain Severity variable, none of our data was normally distributed. Consequently, we used robust analyses (i.e., Spearman's rho, mediation with bootstrapped sampling) to accommodate for potential normality violations. Sequential mediations using PROCESS macro (Model 6, 95% BCI, 5,000 bootstrapped samples) were conducted to evaluate the indirect effect of discrimination on pain outcomes, and vice versa. We chose age, gender, race (Black vs. White), education (some vs. no college), income level (below or above 30,000), and medication status (taking vs. not taking medications) as covariates. Prior literature states that disparities in pain vary based upon these sociodemographic characteristics, and behavioral patterns such as using prescription or over the counter medications for pain or related conditions (79–82). All categorical variables were coded as 0 and 1 for inclusion in analyses. Stress, depressive and insomnia symptoms all independently served as mediators.

## Results

### Participant characteristics

In our sample, 62% (*N* = 129) of our participants identified as Black/African American, and 38% (*N* = 79) identified as White/Caucasian. The percentage of women in our sample was 55.3% (*N* = 115), and 44.7% (*N* = 93) of our participants identified as men. The average age for this sample was ∼45 years old, and the average income ranged from $30,00–$34,999. Moreover, 41.7% (*N* = 83) of our participants had lower incomes (e.g., below 30,000), and 58.3% (*N* = 116) had higher incomes (e.g., above 30,000). Additionally, 77.9% (*N* = 162) of our sample had some college experience, followed by 22.1% (*N* = 46) of those with no college experience. Almost half of our participants (*N* = 89) were currently taking non-steroidal anti-inflammatory drugs (*N*SAIDS), and 33.7% (*N* = 70) of our participants had high blood pressure. Of the 208 participants, over half had moderate pain severity 52.5% (*N* = 106), followed by 20.8% (*N* = 42) with severe pain, 17.8% (*N* = 36) with mild pain, 5.9% (*N* = 12) with very severe pain, 2.5% with very mild pain, and 1 person who marked “no pain” on the BPI-SF. Cutoff scores from the ISI also demonstrated that 33.7% (*N* = 70) of our participants had subthreshold insomnia symptoms, followed by 29.3% (*N* = 61) with moderate symptoms, and 10.1% (*N* = 21) with severe insomnia. Importantly, 26.9% of our participants did not meet cutoff scores for clinically significant insomnia. A vast majority (46.8%, *N* = 101) of our participants met the cutoff criteria for clinically significant depressive symptoms. Full sociodemographic and clinical characteristics are shown in Tables 1, 2, and responses to the everyday discrimination scale will be shown in Supplementary Figure S1.

**Table 2:** Clinical groups (*N* = 208).

Group	*N* (%)
Insomnia	
None	56 (26.9%)
Subthreshold	70 (33.7%)
Moderate	61 (29.3%)
Severe	21 (10.1%)
Pain Severity*	
None	1 (0.5%)
Very Mild	5 (2.5%)
Mild	36 (17.8%)
Moderate	106 (52.5%)
Severe	42 (20.8%)
Very Severe	12 (5.9%)
Depression	
Clinically Significant	101 (46.8%)
Not Clinically Significant	107 (51.4%)

* Has missing data.

### Spearman's Rho correlations

As seen in Table 3, greater discriminatory experiences were associated with male gender (*ρ* = −.20, *p* = .004), greater perceived stress (*ρ* = .31, *p* < .001), symptoms of depression (*ρ* = .38, *p* < .001), and insomnia (*ρ* = .26, *p* < .001), higher pain severity (*ρ* = .23, *p* < .001), and greater pain interference (*ρ* = .25, *p* < .001). Greater perceived stress was associated with younger age (*ρ* = −.18, *p* = .009), greater symptoms of depression (*ρ* = .35, *p* < .001), and insomnia (*ρ* = .35, *p* < .001), higher pain severity (*ρ* = .19, *p* = .005), and greater pain interference (*ρ* = .28, *p* < .001). Symptoms of depression were associated with having a lower income (*ρ* = .20, *p* = .004), greater insomnia symptoms (*ρ* = .62, *p* < .001), higher pain severity (*ρ* = .43, *p* < .001), and greater pain interference (*ρ* = .57, *p* < .001). Lastly, insomnia symptoms were associated with higher pain severity (*ρ* = .45, *p* < .001) and interference (*ρ* = .57, *p* < .001). While most correlations were moderate (less than 0.5), the associations between sleep, mood, and pain outcomes were stronger (<0.6).

**Table 3:** Spearman's rho correlations.

Variable	1	2	3	4	5	6	7	8	9	10	11	12
1. Race	—											
2. Gender	.11	—										
3. Age	.11	−.07	—									
4. Income	.17*	−.13	.03	—								
5. Education	.10	−.05	−.05	.26**	—							
6. Meds	−.19**	.09	.21**	−.05	−.04	—						
7. TEDS	.07	−.20**	−.05	.10	.13	−.12	—					
8. PSS	−.03	.07	−.18*	.01	.11	.007	.31**	—				
9. CESD	−.01	−.07	−.12	.20**	.11	.07	.38**	.35**	—			
10. ISI	.04	.11	.03	.10	.04	.002	.26**	.35**	.62**	—		
11. BPI-S	.28**	−.02	.17*	.24**	.23**	.08	.23**	.19**	.43**	.45**	—	
12. BPI-I	.15*	−.03	.21*	.22**	.21**	.13	.25**	28**	.57**	.57**	.79**	—

Race coded: 0 = White/Caucasian, 1 = Black/African American; Gender coded: 0 = Men, 1 = Women; Income coded: 0 = lower income, 1 = higher income; Education coded: 0 = some college experience, 1 = no college experience; Medications coded: 0 = not currently taking medications, 1 = currently taking medications; TEDS, everyday discrimination scale; PSS, perceived stress scale; CESD, center for epidemiological studies depression scale; ISI, insomnia severity index; BPI-S, pain severity subscale; BPI-I, pain interference subscale.

* *p* ≤ .05.

** *p* ≤ .01.

### Preliminary analyses: hierarchical regressions

To examine the associations among psychosocial risk factors and self-reported pain severity, hierarchical linear regressions were conducted. Sociodemographic variables (age, race, gender, income level, some or no college experience, medications) were entered into Model 1, explaining 15% of the variance in pain severity (F = 5.40, *p* < .001). Specifically, identifying as Black (*β* = .23, *p* = .001), having a lower income (*β* = .16, *p* = .02), and no college experience (*β* = .15, *p* = .03) were associated with greater pain severity. When added to the model, psychosocial risk factors contributed an additional 24% of variance in self-reported pain severity (F = 11.65, *p* < .001). Specifically, identifying as Black (*β* = .23, *p* < .001), greater depressive symptoms (*β* = .18, *p* = .02) and greater insomnia symptoms (*β* = .29, *p* < .001) were associated with greater self-reported pain severity. While overall significant (F = 10.54, *p* < .001), Model 3 didn't contribute additional variance to self-reported pain severity (*p* = .95). All regression coefficients will be shown in Table 4.

**Table 4:** Hierarchical regression (pain severity).

Variable	Step 1				Step 2				Step 3					
	b	SE	*β*	*p*	b	SE	*β*	*p*	b	SE	*β*	*p*	R2	ΔR2
Step 1													.15	
Age	.01	.01	.09	.20	.01	.01	.11	.08	.01	.01	.10	.08		
Race	1.12	.34	.23	.001	1.10	.29	.23	<.001	1.10	.29	.23	<.001		
Gender	−.18	.33	−.03	.58	−.19	.29	−.04	.51	−.19	.29	−.04	.52		
Income	.77	.34	.16	.02	.37	.29	.07	.20	.37	.29	.07	.20		
Education	.90	.41	.15	.03	.70	.35	.12	.05	.70	.36	.12	.05		
Medications	.82	.42	.14	.05	.67	.37	.11	.07	.67	.37	.11	.07		
Step 2													.39	<.001
TEDS					.02	.01	.07	.29	.02	.05	.08	.64		
PSS					.04	.04	.06	.31	.04	.04	.06	.32		
CESD					.04	.01	.18	.02	.04	.01	.19	.03		
ISI					.09	.02	.29	<.001	.09	.02	.29	<.001		
Step 3														
INT									−.01	.30	−.01	.95	.39	.95

Race coded: 0 = White/Caucasian, 1 = African American; Gender coded: 0 = Men, 1 = Women; Income coded: 0 = lower income, 1 = higher income; Education coded: 0 = some college experience, 1 = no college experience; Medications coded: 0 = not currently taking medications, 1 = currently taking medications; TEDS, everyday discrimination scale; PSS, perceived stress scale; CESD, center for epidemiological studies depression scale; ISI, insomnia severity index; INT, interaction term between race and TEDS.

Additional analyses were conducted to examine the associations between psychosocial risk factors and pain interference. Model 1 included demographic covariates and explained 14% of the variance in self-reported pain interference (F = 5.01, *p* < .001). Specifically, identifying as Black (*β* = .17, *p* = .02), having lower income (*β* = .16, *p* = .02), no college experience (*β* = .16, *p* = .02), and taking medications (*β* = .16, *p* = .02) were associated with greater pain interference. Model 2 explained an additional 36% of variance in self-reported pain interference (F = 18.15, *p* < .001), such that older age (*β* = .14, *p* = .01), identifying as Black (*β* = .18, *p* = .001), no college experience (*β* = .11, *p* = .03), and taking medications (*β* = .11, *p* = .04) were associated with greater pain interference. Moreover, greater depressive symptoms (*β* = .33, *p* < .001), and insomnia symptoms (*β* = .32, *p* < .001) were associated with greater pain interference. These findings remained significant in Model 3 (F = 16.42, *p* < .001). All regression coefficients will be shown in Table 5.

**Table 5:** Hierarchical regression (pain interference).

Variable	Step 1				Step 2				Step 3					
	b	SE	*β*	*p*	b	SE	*β*	*p*	b	SE	*β*	*p*	R2	ΔR2
Step 1													.14	
Age	.02	.01	.13	.06	.02	.01	.14	.01	.02	.01	.14	.01		
Race	.87	.37	.17	.02	.92	.28	.18	.001	.92	.28	.18	.002		
Gender	−.05	.36	−.01	.87	−.17	.28	−.03	.55	−.17	.28	−.03	.54		
Income	.84	.37	.16	.02	.31	.29	.06	.27	.31	.29	.06	.27		
Education	1.03	.45	.16	.02	.72	.34	.11	.03	.71	.35	.11	.04		
Medications	1.01	.45	.16	.02	.72	.36	.11	.04	.72	.36	.11	.04		
Step 2													.50	<.001
TEDS					−.003	.01	−.008	.89	−.01	.05	−.04	.80		
PSS					.03	.04	.05	.36	.03	.04	.05	.36		
CESD					.07	.01	.33	<.001	.07	.01	.33	<.001		
ISI					.11	.02	.32	<.001	.11	.02	.32	<.001		
Step 3														
INT									.06	.29	.03	.82	.50	.82

Race coded: 0 = White/Caucasian, 1 = African American; Gender coded: 0 = Men, 1 = Women; Income coded: 0 = lower income, 1 = higher income; Education coded: 0 = some college experience, 1 = no college experience; Medications coded: 0 = not currently taking medications, 1 = currently taking medications; TEDS, everyday discrimination scale; PSS, perceived stress scale; CESD, center for epidemiological studies depression scale; ISI, insomnia severity index; INT, interaction term between race and TEDS.

### Sequential mediation analysis: pain severity

**Figure 1:**
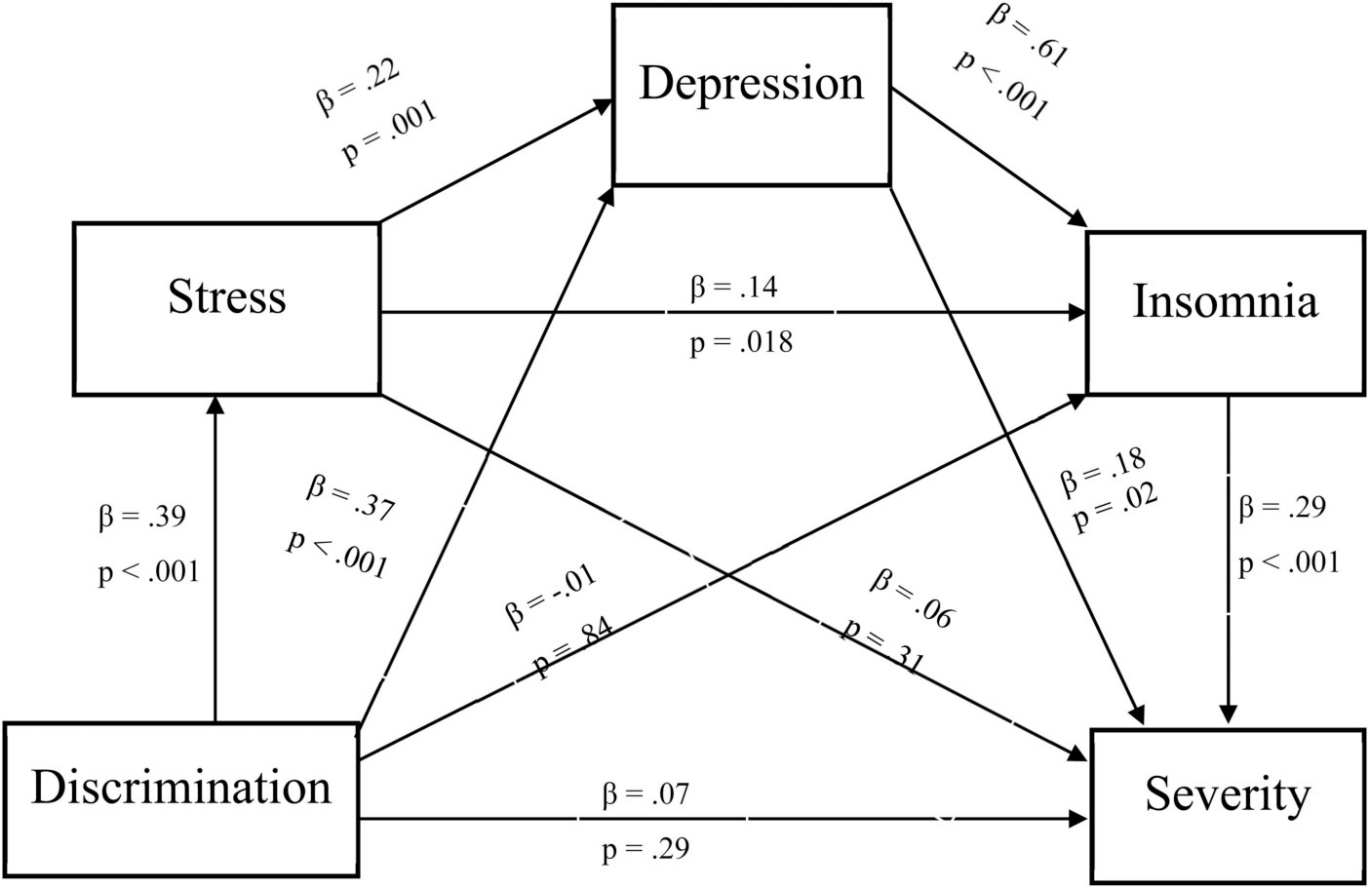
Indirect effect of daily discrimination on pain severity. Note: *β* = .015, 95% Bootstrap CI = .003 - .030.

As seen in Figure 1, there was a significant indirect effect of discrimination on pain severity through psychosocial risk factors, with a point estimate of.015 (95% CI: .003 to .030). Specifically, experiences of discrimination were associated with greater perceived stress (*β* = .39, *p* < .001), greater perceived stress was associated with greater depressive symptoms (*β* = .22, *p* = .001), greater depressive symptoms were associated with greater insomnia symptoms (*β* = .61, *p* < .001), and greater insomnia symptoms were associated with greater pain severity (*β* = .29, *p* < .001). Full descriptions and coefficients for each individual pathway will be shown in Figure 1.

### Sequential mediation analysis: pain interference

**Figure 2:**
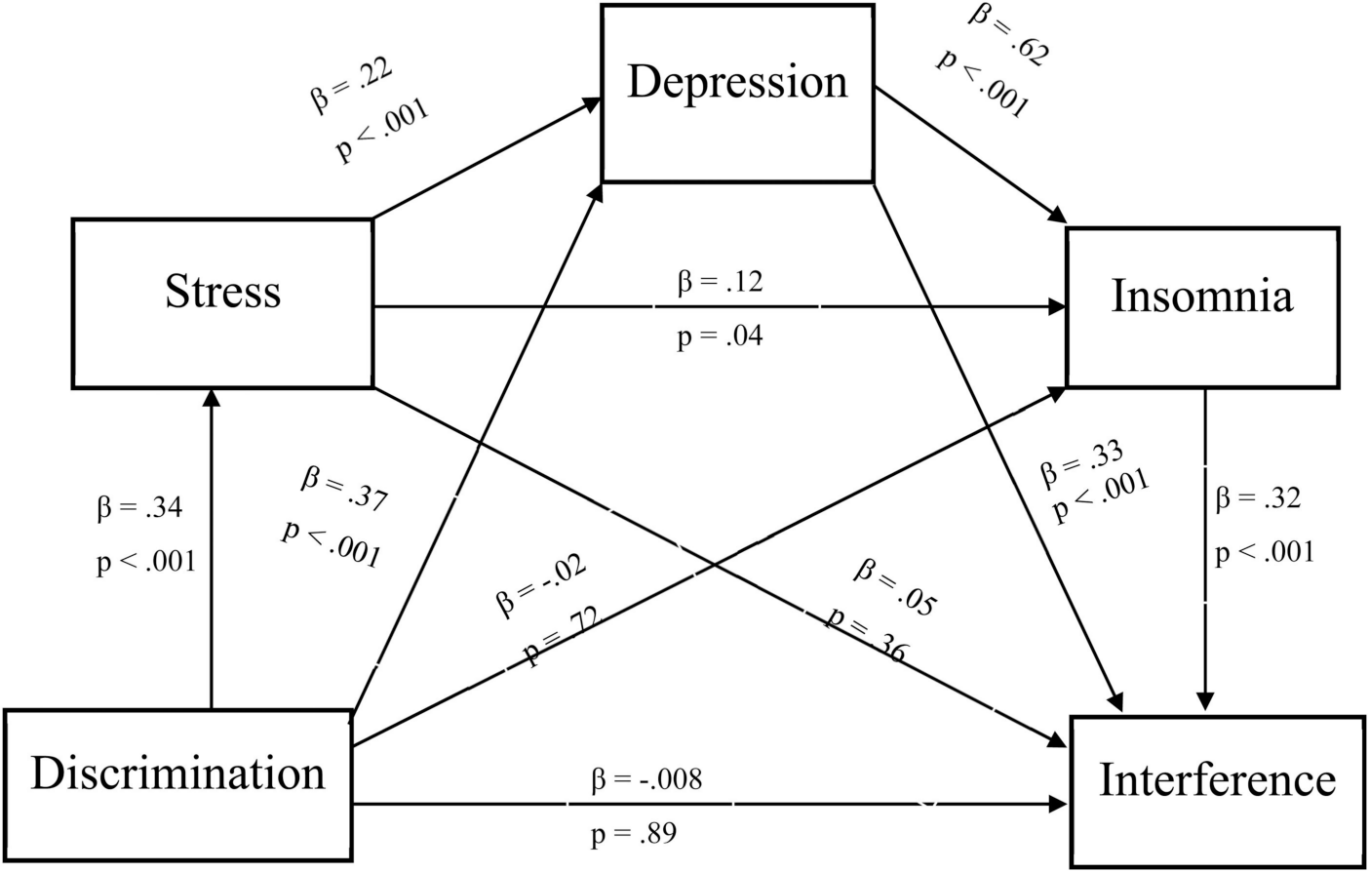
Indirect effect of daily discrimination on pain interference. Note: *β* = .015, 95% Bootstrap CI = .004 - .031.

There was also a significant indirect effect of discriminatory experiences on pain interference via psychosocial risk factors, with a point estimate of .015 (95% CI: .004 to .031). Specifically, greater experiences of discrimination were associated with greater perceived stress (*β* = .34, *p* < .001), greater perceived stress were associated with greater depressive symptoms (*β* = .22, *p* < .001), greater depressive symptoms were associated with greater insomnia symptoms **(***β* = .62, *p* < .001), and greater insomnia symptoms were associated with greater pain interference (*β* = .32, *p* < .001). Full descriptions and coefficients for each individual pathway will be shown in Figure 2.

### Secondary analyses: reverse mediations

**Figure 3:**
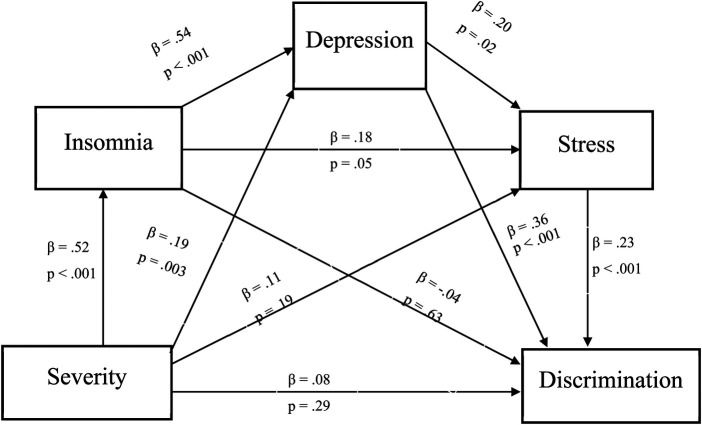
Indirect effect of pain severity on daily discrimination. *Note*: *β =* .014, 95% Bootstrap CI = .001 - .029.

We conducted exploratory analyses to assess the indirect effects of pain severity and interference on experiences of discrimination. There was a significant indirect effect of pain severity on discriminatory experiences via psychosocial risk factors with a point estimate of .014, 95% CI: .001 to .029). Specifically, greater pain severity was associated with greater insomnia symptoms (*β* = .52, *p* < .001), greater insomnia symptoms was associated with greater depressive symptoms (*β* = .54, *p* < .001), greater depressive symptoms was associated with greater perceived stress (*β* = .20, *p* = .02) and greater perceived stress was associated with greater discriminatory experiences (*β* = .23, *p* < .001). Full descriptions and coefficients for each individual pathway will be shown in Figure 3.

**Figure 4:**
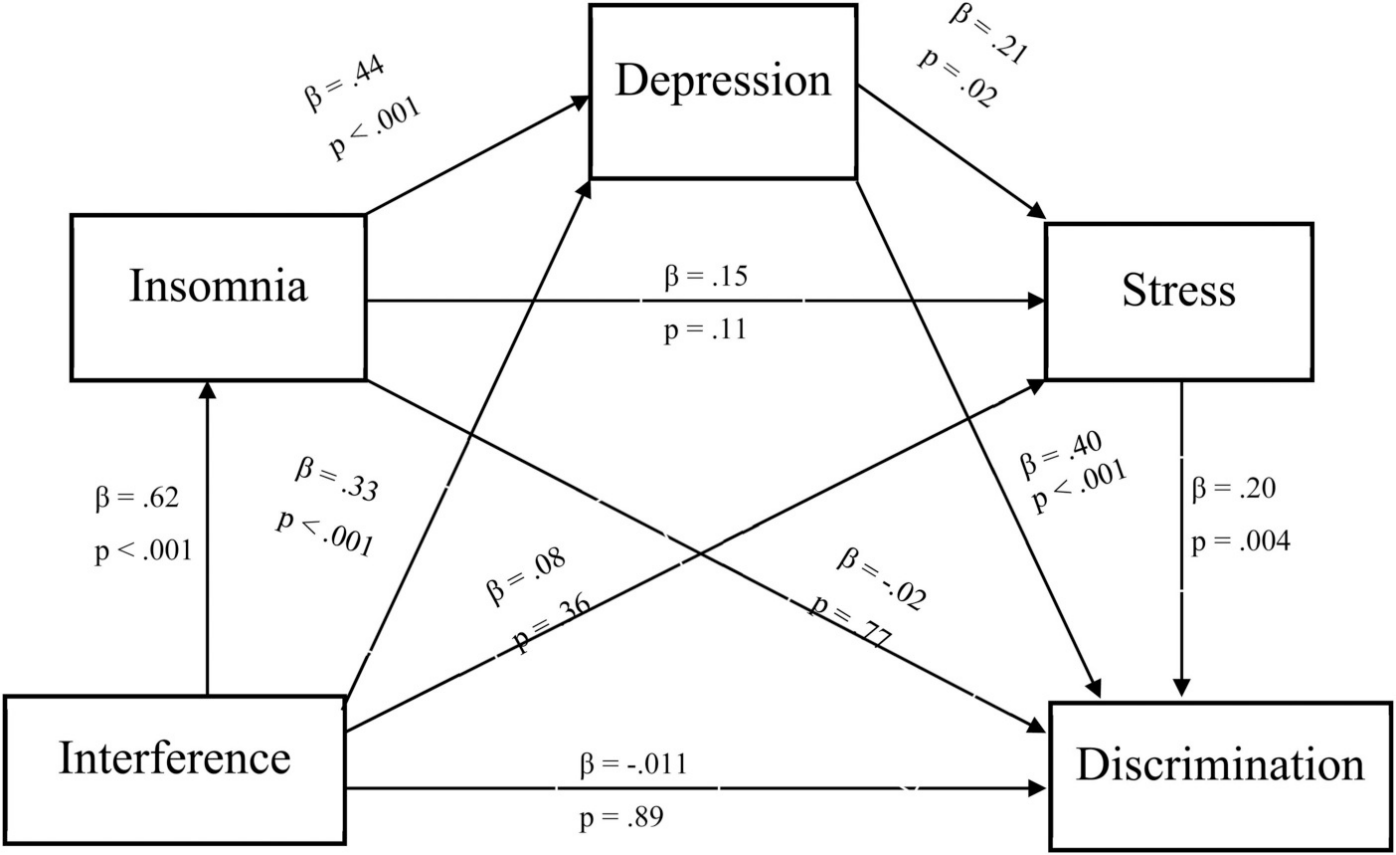
Indirect effect of pain interference on daily discrimination. *Note*: *β =* .012, 95% Bootstrap CI = .0003 - .029.

There was also a significant indirect effect of pain interference on daily discrimination via psychosocial risk factors (point estimate = .012, 95% CI: .000 to .029). Specifically, greater pain interference was associated with greater insomnia symptoms (*β* = .62, *p* < .001), greater insomnia symptoms were associated with greater depressive symptoms (*β* = .44, *p* < .001), greater depressive symptoms were associated with greater perceived stress (*β* = 21, *p* = .02), and greater perceived stress was associated with greater discriminatory experiences (*β* = .20, *p* = .004). Full descriptions and coefficients for each individual pathway will be shown in Figure 4.

## Discussion

### Novelty of study

Based on our research, we found significant associations between discrimination, psychosocial risk factors (e.g., sleep, stress and mood), and pain outcomes in people with cLBP. Additionally, the indirect effect of discrimination on pain severity and interference was significant. These findings align with prior research that suggests an association between discrimination and negative pain-related outcomes, ultimately affecting quality of life, co-morbidity burden, and accelerated mortality (16, 83, 84). Moreover, our regressions showed that certain demographic (e.g., Black race, lower education) and behavioral (e.g., taking medications, depression and insomnia symptoms) characteristics were associated with worse pain outcomes, which aligns with previous research (48, 53, 80, 81, 85). Lastly, mediation analyses showed that the associations between daily discrimination and pain outcomes could be partially explained by other psychosocial risk factors (stress, depression and insomnia)—a finding that hasn't been explored and sets the foundation for future intervention research and health initiatives. Taken together, this manuscript highlights that having cLBP introduces discrimination, which may contribute to negative physical and emotional aspects associated with worse pain outcomes (i.e., pain severity, mood, physical functioning). As seen above, discrimination impacts perceptions of stress, depression and insomnia, and these effects lead to greater perceptions of pain severity and interference (44, 84, 86, 87). In turn, experiencing chronic pain may present as a precursor for co-morbid symptoms like insomnia and depression, thus introducing a vulnerability to stigma and related discrimination (53, 83). In our study, these relationships persisted even after controlling for demographics (i.e., age, gender, race, income and education, medications). This suggests that in addition to demographic disparities in pain and mental health, there is an association between discrimination, pain, and quality of life that deserves a further look (12, 88, 89). Together, these findings demonstrate the robust and negative impact of discrimination and pain on multiple levels of health and functioning (84, 89–91). Overall, we provide novel evidence of potential therapeutic targets (e.g., stress-reduction, positive psychology, trauma informed care) for intervention, while also contributing to prior literature aiming to improve pain and physical functioning in people with cLBP, which we will elaborate on further (92, 93).

### Sequential mediations: the role of psychosocial risk factors

Although both mediations were significant, the indirect effects of discrimination on pain severity and interference were slightly stronger. This finding aligns with prior work suggesting that consistent experiences of ostracism heighten the severity of pain, as well as how pain interferes with daily life (i.e., pain perception, disability) (33, 94). Importantly, the associations between discrimination and pain outcomes were fully explained by psychosocial mediators, such as stress, depressive symptoms, and poor sleep. To elaborate, experiences of discrimination can often be deemed stressful, and theories such as the Social Pain theory, and the Transactional Model of Stress and Coping support our findings (95–97). Specifically, the social pain theory suggests that experiences of ostracism ignite some of the same negative emotions that are induced during physical threat or injury—thus contributing to a heightened pain perception and experience (96, 98). On the contrary, the transactional model of stress and coping suggests that stress negatively impacts health and coping behaviors when a person perceives that their individual resources and capacities are consistently outweighed by their daily demands (95). For people with cLBP, this experience can be common due to the taxing demands of having a debilitating health condition, amongst other intersectional identities and pressures (i.e., Black race, lower socioeconomic status, taking pain or related medications) (99). Taken together, the experience of discrimination has underlying health effects that warrants further attention for the development of stress-informed interventions and care in people with cLBP.

In addition to appraisals of stress, prior literature suggests that discriminatory experiences can introduce feelings of helplessness, and rumination—both symptoms of depression that can be detrimental (44–46, 100). Importantly, nearly half of our sample met diagnostic criteria for clinically significant depression. Although not measured directly, prior work has shown that experiences of stigma and discrimination can be internalized, leaving people to consistently cope and appraise the situation, and throwing off daily patterns (i.e., rumination, poor sleep) (44, 84, 101, 102). Consequently, symptoms of insomnia can arise, and this is heightened in people with pain conditions for a variety of reasons (e.g., shared neural circuitry, coping patterns, demographics) (47, 48, 89, 103). Future research could involve using the following strategies to address these areas: (1) cognitive or brief behavioral therapy for insomnia (CBT-I, BBT-I) to improve sleep and mood, (2) resilience enhancing interventions (positive psychology, exercise) to improve mental health among minority groups who experience discrimination, (3) talk-therapy and mindfulness-based stress reduction (MBSR) interventions for symptoms of stress and depression, and (4) yoga and spirituality as holistic measures for targeting all factors (93, 104–106). More specifically, a recent study in people with HIV and pain found that the use of Brief Behavioral Therapy for Insomnia (BBT-i) decreased insomnia symptoms and pain interference from pre- to post-intervention (104). This trend has also been replicated in football players, with decreases in both pain severity and interference (107). Positive psychology and music analgesia have also been implicated as successful interventions to reduce stress, pain interference and severity (92, 93, 108). Lastly, mindful meditation and physical activity interventions have been shown to decrease both depression and pain (109, 110). While interventions addressing stigma and discrimination are scarce, future work could involve bias trainings and policy changes that build health equity and equal access to resources (i.e., health education, science communication, pop-up health screenings, food and transportation assistance) with hopes to lessen the burden of systemic and interpersonal marginalization (12).

### Pain and sociodemographic risk factors

A series of regressions showed that identifying as Black, having a lower education, and taking daily medications were also associated with greater pain-related interference. Moreover, identifying as Black was also associated with a greater severity of pain. While previously noted, these findings shed light upon the economic, physical, and mental risk that minoritized groups with pain face daily (99). For example, prior work states that living with one or more stigmatizing identities (e.g., intersectionality) can influence the trajectory of your health—especially for people with chronic pain (12, 100). Moreover, a vast amount of literature has shown that those with lower socioeconomic statuses have an additive burden with pain management (access to medication and resources, health insurance) that often exacerbates their pain experience (82, 111, 112). Coupled with the daily pressures of having chronic pain, these additive layers of complexity can introduce a range of daily complications, and could be intervened with by introducing more resources, health education interventions, and mutual aid initiatives (93, 113). For example, if a person with lower income is taking pain medications, a health education session could determine the difference between adaptive or maladaptive coping patterns. In addition to this, mutual aid initiatives could bridge the gap between pain-related disability and pain management in those with less tools and disadvantaged backgrounds. In summary, future research will extend beyond psychological risk factors to enhance pain management and treatment among those from disadvantaged backgrounds.

### Clinical implications

In addition to novel advances in research, our study has implications that can be applied clinically. For example, knowledge of the co-occurrence among stress, depression and insomnia symptoms can inform more holistic approaches to clinical care. Therefore, instead of focusing on the physical representation of the patient, physicians can move towards individualized, person-centered care (114). Non-pharmacological techniques such as breathing exercises, journaling, and physical activity have all shown to decrease symptoms of stress, pain and depression (115–118). Moreover, spending additional time assessing the patient's daily routines and habits could really inform their treatment recommendations. For example, if a patient with cLBP presents with severe pain, doctors can begin to assess the upstream or underlying reasons (e.g., asking how they sleep on a regular basis, what are their daily stress levels, how is their mood?) instead of providing prescription medications alone. Treating the underlying root of the condition could help lessen the burden and severity of pain, while also giving patients tools that could be translated into their daily lives (e.g., breathing activities for stress reduction, improving sleep hygiene). Most importantly, experiences of healthcare and interpersonal discrimination could be addressed by emphasizing patient advocacy and resilience (10, 119). While discriminatory experiences are hard to control, leveraging individual resources, building barriers of resilience, and community (i.e., social support) can circumvent the internalized thoughts that often affect the perception and experience of pain in minorities.

### Study limitations

This study possesses several limitations that warrant discussion. First, this study was cross-sectional and hypothesis generating in nature. Specifically, our sequential mediation models were designed to test the possibility of an indirect association between daily discrimination and pain outcomes through psychosocial risk factors. Because sessions were only separated by one week, we are limited in determining directionality and inference. Thus, results should be interpreted with caution due to the limitations of time constraints on directionality. To circumvent this, we conducted backwards mediations to assess indirect effects from both discrimination and pain outcomes. Future research will incorporate a longitudinal design to assess the true directionality (i.e., causal models) and strength of associations. Secondly, our study used single-site recruitment and involved a high level of compensation (e.g., $400) that could introduce possible selection bias. Future work could incorporate these measures on a larger scale, using multi-site recruitment strategies to further generalize our findings. Third, we examined cumulative experiences of discrimination; however, discriminatory experiences are often shaped by historical and cultural contexts, due to power differentials and oppression affecting some groups more than others; thus, future studies should include larger and diverse samples to examine how these relationships vary based on sub-groups. We did not do group comparisons based on sociodemographic characteristics, and we also didn't have measures of stress reactivity despite the mention in our introduction (i.e., HPA axis dysregulation). However, like previous studies, we wanted to first examine the associations between stressful experiences, pain, and mental health that haven't been shown in people with cLBP. Future research will incorporate objective biomarkers of stress, and measures of differences based on demographic characteristics to get a fuller picture of discrimination and pain outcomes. Lastly, the possibility of pain outcomes being explained by other confounders (i.e., BMI, pain duration) wasn't included in this study, and our sample size was relatively small. Future work will incorporate a more extensive approach to understanding pain and pain management, as well as using a larger sample size to promote generalizability to the larger cLBP population.

### Study strengths, conclusions, and future research

As the first study testing these sequential models in a cLBP sample, our results highlight potential avenues that explain, in part, the associations between daily discrimination and pain outcomes. These pathways highlight the need to evaluate how discrimination influences additional psychological variables (i.e., stress, depression) and sleep functioning, which often co-exist with and exacerbate the pain experience. Our findings also provide evidence for vulnerability to discriminatory experiences in people with cLBP, and how these experiences may be internalized through depressive symptoms and stress. Moreover, our findings represent the importance of recognizing sociodemographic characteristics in clinical interventions and study design (e.g., Black race, lower SES, taking medications). This is the first study to elaborate on these indirect effects, and future studies should target these mechanistic pathways for intervention and treatment strategies (e.g., longitudinal design, larger sample, MBSR, BBTi, mutual aid and bias trainings), as well as narrow down on minority groups (e.g., racial minorities, LGBTQ+, older adults, people who use substances, women).

## Data Availability

The raw data supporting the conclusions of this article will be made available by the authors, without undue reservation.
