# Supplementary material for: Examining associations among daily discrimination, psychosocial risk factors, and pain outcomes in people with chronic low back pain
Source: Front Pain Res (Lausanne). 2026 Jan 12;6:1531187. doi: 10.3389/fpain.2025.1531187 (PMC12833447; doi:10.3389/fpain.2025.1531187)
Supplement: Supplementary file 1 [file Table1.docx]

**Table 1: Participant Characteristics (N = 208)**

| Variable (Mean, SD or N, %) |  |
| --- | --- |
| **Demographics** | **(Mean, SD or N, %)** |
| Age | 44.94 (14.37) |
| Gender, N (%)  Men | 93 (44.7%) |
| Women | 115 (55.3%) |

Race, N (%)

African American/Black 129 (62.0%)

Caucasian/White 79 (38.0%)

Income*

Lower income (below 30,000) 83 (41.7%)

Higher income (above 30,000) 116 (58.3%) Education

Some college experience 162 (77.9%)

No college experience 46 (22.1%)

# Medications & Comorbidities

Medications*

Taking medications 165 (81.3%)

Not taking medications 38 (18.7%)

Medication Type*

| Opioids | 18 (8.7%) |
| --- | --- |
| NSAIDs | 89 (42.8%) |
| Muscle relaxers | 19 (9.1%) |
| Antidepressants | 33 (15.9%) |
| Neuroleptics | 3 (1.4%) |
| Benzodiazepines | 6 (2.9%) |
| Vitamins | 35 (16.8%) |
| Botanicals | 5 (2.4%) |
| Blood pressure medication | 48 (23.1%) |
| Diabetic medication | 14 (6.7%) |
| Allergy medication | 10 (4.8%) |
| Thyroid medication | 9 (4.3%) |
| Cholesterol medication | 10 (4.8%) |
| Non-NSAID Pain medication | 28 (13.5%) |
| Anticonvulsant medication | 24 (11.5%) |
| Asthma medication | 7 (3.4%) |
| Comorbidities* | |
| High blood pressure | 70 (33.7%) |
| Heart disease | 3 (1.4%) |
| Cancer | 0 |

| Diabetes (Hba1c > 7%) | 23 (11.1%) |
| --- | --- |
| Ankylosing Spondylitis | 1 (0.5%) |
| Infection | 0 |
| Parkinson's Disease | 0 |
| Multiple Sclerosis | 0 |
| Epilepsy | 1 (0.5%) |
| Syndromic obesity | 0 |
| Stroke | 0 |
| Seizure | 1 (0.5%) |
| Rheumatoid arthritis | 3 (1.4%) |
| Lupus erythematosus | 0 |
| Fibromyalgia | 0 |
| Major depression/BPD | 19 (9.1%) |
| Other mental health condition | 36 (17.3%) |
| HIV | 0 |
| **Survey Characteristics** |  |
| TEDS – Discrimination, Mean (SD)* | 9.78 (8.70) |
| PSS – Stress, Mean (SD) | 21.61 (3.88) |
| CES-D – Depressive Symptoms, Mean (SD) | 16.63 (10.64) |
| ISI – Insomnia Symptoms, Mean (SD) | 12.32 (6.98) |
| BPI-SF Pain Severity Mean (SD) | 4.47 (2.35) |
| BPI-SF Pain Interference Mean (SD) | 3.10 (2.46) |

Note: BPI-SF = brief pain inventory short form; CES-D = center for epidemiological studies depression scale, ISI

= insomnia severity index: PSS = perceived stress scale; TEDS = the everyday discrimination scale; * = has missing data
